# Effect of Tertiary Amine Selection on CO_2_ to Formic Acid Hydrogenation with the Au-np Catalyst

**DOI:** 10.1021/acs.iecr.4c04902

**Published:** 2025-04-10

**Authors:** Anouk W.N. de Leeuw den Bouter, Luca M.P. Meijer, Larissa Brito, Adeline Miquelot, Pierre Olivier, John van der Schaaf

**Affiliations:** 1 Sustainable Process Engineering, Chemical Engineering and Chemistry, Eindhoven University of Technology, Het Kranenveld 14, Eindhoven 5612 AZ, The Netherlands; 2 Lab Hydrogen, ENGIE Lab CRIGEN, 4 Rue Josephine Baker, N/A, Stains 93240, France; 3 Lab Biogas, Biomass and Waste, ENGIE Lab CRIGEN, 4 Rue Josephine Baker, N/A, Stains 93240, France

## Abstract

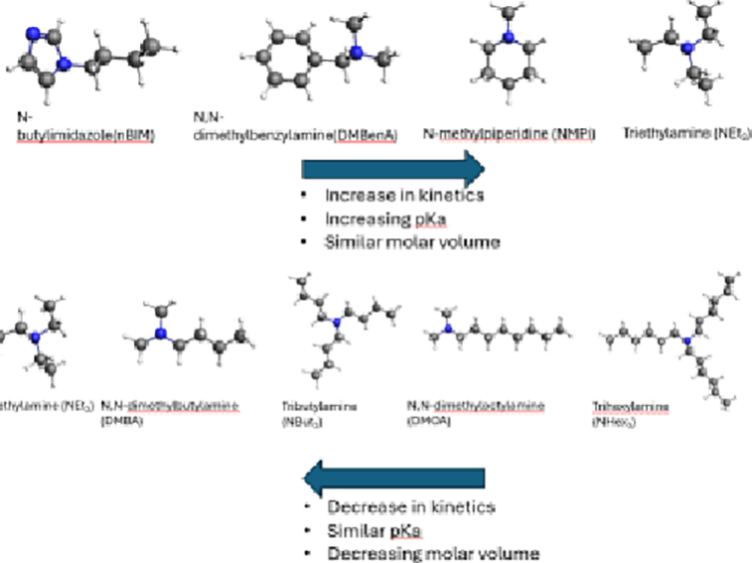

Over the past decades, the production and storage of molecular hydrogen has been identified as a key solution to the current global warming crises. Here, formic acid has gained considerable traction as a possible liquid organic hydrogen carrier. One of the possible production methods is the direct hydrogenation of CO_2_. However, the reaction is highly unfavorable from a thermodynamic point of view and can be made slightly favorable using tertiary amines. The addition of tertiary amines leads to adduct formation, which drives the reaction toward the product side through reduction of the formic acid activity in the bulk liquid. However, currently, the competitiveness of the process is severely hindered by the challenging and energy-intensive formic acid purification due to the formation of azeotropes between formic acid and the low-boiling tertiary amines. Additionally, the reaction rates are hampered by the limited solubility of the adduct and nonbinding amine leading to the necessity of additional solvents. Steric hindrance and the p*K*_a_ of the tertiary amine were identified as key parameters influencing both the observed kinetic rates and the CO_2_ conversion. The usage of solventless polar amines such as diethylethanolamine allowed for FA productivity up to 5× that of the benchmark triethylamine system. Catalyst deactivation of the Au/TiO_2_ catalyst was observed for all amines studied within this work, and the deactivation mechanism was shown to be sintering of the Au nanoparticles with no significant leaching, morphological changes, or oxidation of the Au species observed.

## 1 Introduction

Nowadays, global energy demands have skyrocketed as a consequence of mass industrialization and globalization. This has resulted in significant energy consumption rates, with the global energy demand in 2021 being 576 TJ^1^ and expected to increase to 672 TJ in 2050.^2^ The major energy sources are fossil fuels such as oil, coal, and natural gas, with renewables only making up to 4.3% of the total energy consumption in 2021.^2^ Fossil fuels will not only be depleted within the near future but also result in the emission of greenhouse gases such as CO_2_.^3^ In order to prevent further human-induced climate change, future emissions need to be drastically reduced and the urge for more renewable fuels is omnipresent.^4,5^ One such an alternative fuel is hydrogen, which has been widely recognized as a clean universal energy carrier with near zero greenhouse gas emissions.^6−8,11^

However, hydrogen has a low boiling point and low volumetric energy density under standard conditions,^9,10^ resulting in the necessity of compression or liquefaction to allow for economical and safe transport and storage.^9^ Alternative storage options include low-temperature physisorption on high-surface-area materials,^12,13^ chemisorption at ambient conditions on complex hydrides,^12^ or direct chemical transformation into fuels such as methanol and formic acid.^13,14^ Despite promising results obtained for physisorption and chemisorption techniques in previous works,^15−17^ the hydrogen storage capacity remains low compared to direct chemical storage in the form of liquid organic hydrogen carriers (LOHC).^18−21^ A promising example of an LOHC has been identified to be formic acid due to its 100% theoretical atom efficiency,^14^ highly reversible nature at low temperatures,^22^ high chemical stability at ambient conditions, and low toxicity and flammability, combined with a volumetric energy density of 53.4 g/L.^23^

The direct hydrogenation of carbon dioxide toward formic acid within the gaseous phase is thermodynamically unfavorable (Δ*G*^0^ = 32.8 kJ/mol).^24,25^ The addition of solvents results in an exergonic reaction^24,25^ that can be shifted to the product side using bases or amines.^26,27^ While such systems have shown high catalytic activity, formic acid purification is energy-intensive due to the necessity of acidification or amine-exchange reactions under harsh conditions.^28,29^ The amine-exchange is required due to the employment of low-boiling tertiary amines, resulting in stable azeotropes.^30^ Previous works have shown the amine-exchange reaction to be highly energy-intensive due to significant steam input required within distillation to provide sufficient energy to break the formic acid–tertiary amine bond.^28,29^

Several strategies have been investigated to perform the reaction under base-free, neutral circumstances.^27,31−34^ Common strategies include the usage of ionic liquids as solvents/buffers^27,34^ and the immobilization of amines or ionic liquids onto solid carries,^31,32,34^ often combined with active hydrogenation metals.^35,36^ Typically, such systems operate under high H_2_ and CO_2_ partial pressures resulting in supercritical conditions^37^ and the total system activity obtained is significantly lower compared to basic conditions.^31^

While the economic competitiveness of the direct hydrogenation reaction performed under basic condition is currently hampered by the challenging and energy-intensive separation,^28^ the TOFs obtained are significantly higher compared to base-free systems.^32^ Previous works have shown the employed tertiary amine to be of significant influence on the observed kinetic rates.^25,31,41^ Loges et al. have studied the influence of the tertiary amine performing the formic acid decomposition reaction on 5/2 molar ratio adducts using a homogeneously catalyzed batch process. TOFs up to 445 h^–1^ were observed after 2 h using asymmetrical tertiary amines such as dimethyloctylamine at 40 °C.^39^ Cao et al. performed a similar study, revealing promising results using a heterogeneous gold supported on amphoteric zirconia catalyst showing a TOF up to 1166 h^–1^ for an FA-dimethylethanolamine system at 60 °C.^23^ Recently, Lee et al. studied the hydrogenation of CO_2_ toward formic acid as a reactive CO_2_ capture process based on the formation of bicarbonates using water as a solvent under supercritical conditions. Based on a heterogeneous Ru/bpyTNCTF catalyst, formic acid production was performed in a batch reactor, showing clear correlation between the p*K*_a_ of the amine, steric hindrance, and CO_2_ conversion rates.^38^

Previous work^40^ has shown that the inability of formic acid–tertiary amine adducts to dissolve in free amine results in formic acid remaining confined in the catalyst pores, showing the necessity for the usage of additional solvents. Within this work, 1-decanol is used as a solvent for all trialkylamines studied. Triethylamine is regarded as the state-of-the-art amine due to numerous works reporting promising results^15,28^ and thus used as a reference. However, triethylamine has been shown to form a stable azeotrope with formic acid at 27 mol % formic acid,^30^ rendering direct thermal splitting intractable. While low-boiling tertiary amines such as triethylamine result in azeotrope formation, longer hydrocarbon chain length amines such as trihexylamine do not.^25^

In order to overcome the limitations posed by the employment of low-boiling tertiary amines, this work aims to study the influence of the nature of the tertiary amine used to shift the equilibrium to the product side on CO_2_ conversion rates and product selectivity using alcohol-based solvents in nonsupercritical conditions using a Au/TiO_2_ catalyst. The Au/TiO_2_ catalyst was selected as previous works revealed the catalyst to be stable for extended periods of time (37 days) under supercritical conditions, allowing for a TON of 18,040.^41^

Previous research revealed that the formic acid binding capacity of tertiary amines is independent of the hydrocarbon chain length.^42^ Therefore, this allows for the usage of a wide variety of tertiary amines. Amines were selected based on their boiling point, steric hindrance, p*K*_a_, and solubility with alcohol-based solvents and formic acid. Following these criteria, 15 amines were selected having a wide range of p*K*_a_ values and molecule sizes. To allow for an initial screening of kinetic rates, the much faster formic acid decomposition reaction was performed in a batch reactor using a Au/TiO_2_ catalyst. Following the selection of the most promising amines in terms of FA conversion rates, high-pressure fixed bed experiments were executed for the CO_2_ hydrogenation.

## 2 Experimental Materials and Methods

### 2.1 Materials

Au/TiO_2_ cylindrical catalyst pellets (AUROlite) were bought from Strem Chemicals, Inc. (1 wt % Au, *d*_p_ = 2.6 nm). 1-Decanol (>98%), 2-diethylaminoethanol (99%), and 1-(*n*-butyl)imidazole (99%) were from Thermo Scientific Chemicals. Formic acid (>99%), triethylamine (>99.5%), methyldiethanolamine (>99%), *N*-methylimidazole (>99%), *N*-methylpiperidine (>99%), *N*,*N*-dimethylbenzylamine (>99%), and tributylamine (>98.5%) were from Sigma-Aldrich. Trihexylamine (>95.0%), *N*,*N*-dimethylbutylamine (>98.0%), *N*,*N*-dimethyloctylamine (>95%), 2-(dimethylamino)ethanol (>99.0%), 4-dimethylamino-1-butanol (*>*98.0%), 6-dimethylamino-1-hexanol (>98%), and 1-(3-dimethylaminophenyl)ethanol (>98%) were from TCI Chemicals. Gases (H_2_ (5.0), CO_2_ (4.5), and Ar (5.0)) were supplied by Linde Gas in bottles.

The chemicals used for H NMR and ICP analysis were acetonitrile-*d*_3_, obtained from Thermo Scientific Chemicals (>99 atom % D) and chloroform-*d* from Sigma-Aldrich (>99.8 atom % D). 1,3,5-Trimethoxybenzene was obtained from TCI Chemicals (>98.0%). A gold standard for ICP was from Fluka TraceCERT Ultra (1002 ppm). Hydrofluoric acid EMSURE (48%), nitric acid (65%) EMPLURA (>99.9%), and hydrochloric acid (37%) a.c.s. reagent were from Merck.

All materials were used as received, handled in air, and used without any additional purification. In order to prevent catalyst deactivation, the catalyst was stored under Ar in a cold and dark environment.

### 2.2 Tertiary Amine Testing

Due to the slow kinetic nature of this reaction and the requirement of significant amounts of the catalyst, it was opted to use the formic acid decomposition reaction as a screening tool on the basis of microscopic reversibility. To allow for an initial assessment of activity, formic acid decomposition reactions were performed in batch reactors using a Au/TiO_2_ catalyst. Prior to reactive testing, the catalyst was reduced offline at 423 K in 5 vol % H_2_/He^43^ at a total flow rate of 50 mLn/min. The catalyst was received in the form of extrudates and thus subsequently crushed and sieved to a particle size of 150–250 μm.

Formic acid decomposition experiments were conducted in glass batch reactors. Since it was not possible to sample from these reactors without disturbing the equilibrium, seven identical vials (N18, Macherey-Nagel, 20 mL, N18 magnetic screw caps) were prepared for each reaction. Each vial contained 10 ± 1 mg of the Au/TiO_2_ catalyst and 1 mL of the reaction mixture. The results were normalized per catalyst mass. The reaction mixture consisted of a tertiary amine, formic acid, and, in the case of nonpolar tertiary amines, 1-decanol as a solvent. The formic acid and amine concentration was 1 mol/L, ensuring that no unbound formic acid was present within the solution. In the case of the OH-substituted amines, no solvents were required as these amines do not show biphasic behavior. The formic acid concentration was kept constant at 1 mol/L, with the tertiary amine used for dilution.

The vials were prepared and subsequently put in an aluminum heating block and placed on a magnetic stirring hot plate (IKA RCT basic). The temperature was controlled by using a PID controller (IKA ETS-D5). The heating block was thermally insulated by using thermal insulation to prevent the formation of radial temperature profiles along the radial direction of the heating block. An image of the setup can be found in the SI (Figure S6).

**Figure 1:**
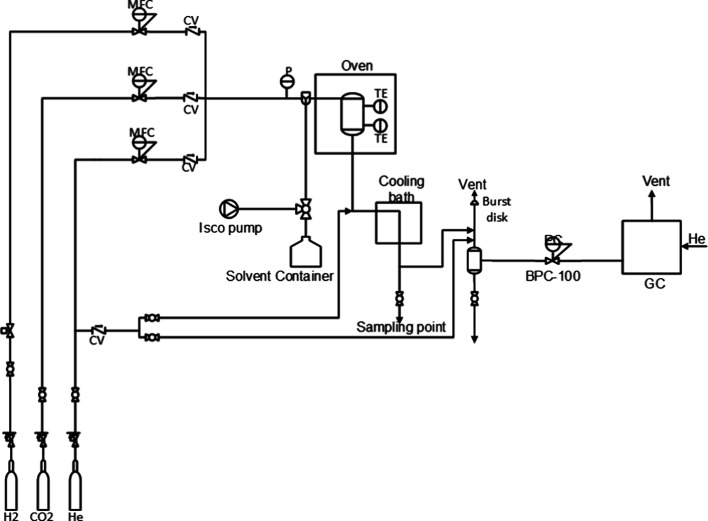
Schematic overview of the setup used for CO_2_ hydrogenation experiments. P, CV, MFC, TE, and BPC indicate pressure transmitters, check valves, mass flow controllers, PT-100, and back pressure controllers, respectively.

CO_2_ hydrogenation experiments were performed at elevated pressure in a stainless-steel packed bed reactor, with a schematic overview given in Figure 1. The reactor consisted of 1/2″ Swagelok VCR connections (internal diameter 10 mm) and a stainless-steel tube with a total length of 10 cm. Metal gaskets with 5 μm pore sizes were used on either end to prevent the catalyst from flushing out of the reactor. The reactor was placed vertically and packed using 1 mm glass beads and quartz wool above and below the catalyst bed to ensure proper liquid–solid contact. The catalyst bed consisted of 2.5 g of the Au/TiO_2_ catalyst (125–180 μm), with no further dilution. The reactor was placed inside an electrical oven, with the temperature monitored using two PT-100s, one placed at the beginning of the catalyst bed and one at the reactor outlet. Liquid was pumped using a syringe pump (Teledyne ISCO 260D). The gas streams were controlled by Bronkhorst MFCs (EL-FLOW Prestige, *F*_max_ = 50 mL_n_/min). To ensure proper gas–liquid mixing, the incoming streams were mixed in a T-piece prior to entering the reactor. The pressure was controlled by a Bronkhorst back pressure controller (EL-PRESS, *F*_max_ = 50 mL_n_/min). A condenser was placed after the reactor to liquefy any condensables, after which the gas and liquid were separated using a gas–liquid separator. Liquid samples were taken from a valve placed behind the cold trap. The gaseous reaction products were analyzed with a compact gas chromatograph (Global Analyzer Solution) equipped with a TCD detector and two packed columns (Molsieve 5A and Rt-Q-BOND). The lower detection limit for CO was established as 2 ppm. This lower detection limit was determined by using calibration bottles supplied by Linde Gas. All reaction tests were performed at a residence time of 15 min (liquid flow rate 1 mL/min, CO_2_ flow rate 1 mL_n_/min, and H_2_ flow rate 1 mL_n_/min). As the desired product was present in the liquid phase, the liquid residence time τ was used and was calculated by
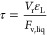
1.1

Here, *V*_r_ represents the total reactor volume, ε_L_ the liquid hold-up, and *F*_v,liq_ the liquid flow rate.

Liquid samples were analyzed by using ^1^H NMR (Bruker, 400 MHz) to determine the chemical composition. Here, 0.5 mL of the deuterated solvent (acetonitrile-*d*_3_ or chloroform-*d*) and 1,3,5-trimethoxybenzene as an internal standard was mixed with 0.1 mL of the sample.

### 2.3 Catalyst Characterization

Transmission electron microscopy (TEM) images of fresh and spent catalysts were acquired by using an FEI CryoTitan transmission electron microscope operating at 300 kV. HAADF-STEM was also acquired on the CryoTitan (300 kV) at room temperature. Samples were prepared by finely crushing the sample, followed by ultrasonic suspension in ethanol and dispersed over a Cu grid with a holey carbon film. The particles were individually identified using image software ImageJ, and the particle size distribution of each catalyst sample was quantified accordingly.

CO pulse chemisorption was performed by using a Micromeritics Autochem II 2920. First, the catalyst was dried and reduced at 300 °C for 60 min under a 40 mL/min 10 vol % H_2_/Ar flow. During this time, the TCD signals were recorded to ensure full reduction. The H_2_ was then purged using a He flow for 60 min. A temperature of −72 °C was reached by submerging the U-shaped glass reaction in a dry ice-isopropanol slurry. For the pulse chemisorption, a 5 vol % CO/He was utilized at a flow rate of 30 mL/min with a pulse time of 2 min. The pulsing was continued until a stable saturation was reached.

Inductively coupled plasma–optical emission spectroscopy (ICP-OES; iCAP PRO ICP-OES; Thermo Scientific) was used to obtain the Au concentrations of the fresh and spent catalysts. Samples were prepared by dissolution of 50 mg of the catalyst in a mixture of concentrated HF (3 mL) and aqua regia (6 mL) at elevated temperatures. After digestion, the samples were filtered and diluted using double distilled water.

X-ray diffraction (XRD) was used for crystal phase identification in the 2θ range 10–120 ° with a Rigaku MiniFlex600 operating with a Ni β-filtered Cu Kα radiation source (40 kV, 30 mA). The scan rate was 0.05 °/min. The diffraction peaks were identified using the JCPDS database.

X-ray photoelectron spectroscopy (XPS) samples were prepared by finely crushing the catalyst, followed by drying *in vacuo* at 50 °C. Measurements were performed using conventional ultrahigh-vacuum XPS (Thermo Scientific K-Alpha, equipped with an Al anode ((Al Kα = 1486.68 eV) monochromatized X-ray source). Samples were prepared by placing the finely crushed catalyst onto double-sided carbon tape. Wide-range survey spectra were recorded using a 200 eV pass energy using 10 scans, while high-resolution core-level spectra were measured using a 50 eV pass energy and 50 scans. The pressure inside the analysis chamber was kept below 8 × 10^–8^ mbar at all times. Surface charging was prevented by using a flood gun (low energy Ar^+^ ions). Energy referencing was performed using the adventitious carbon peak at 284.5 eV. The Au 4f orbital spectra were recorded first to prevent Au reduction due to X-ray exposure.

## 3 Results and Discussion

The results obtained from the FA decomposition reaction are presented first, followed by validation of using the decomposition reaction as a screening tool. The most promising amines were identified and studied for formic acid production in longer time-on-stream experiments in a high-pressure fixed bed reactor. Lastly, the spent catalysts were studied using several solid-state characterization techniques. The absence of internal and external mass transfer limitations was confirmed by using the Mears and Weisz–Prater criteria. Example calculations are included in the SI.

### 3.1 Formic Acid Decomposition

**Figure 2:**
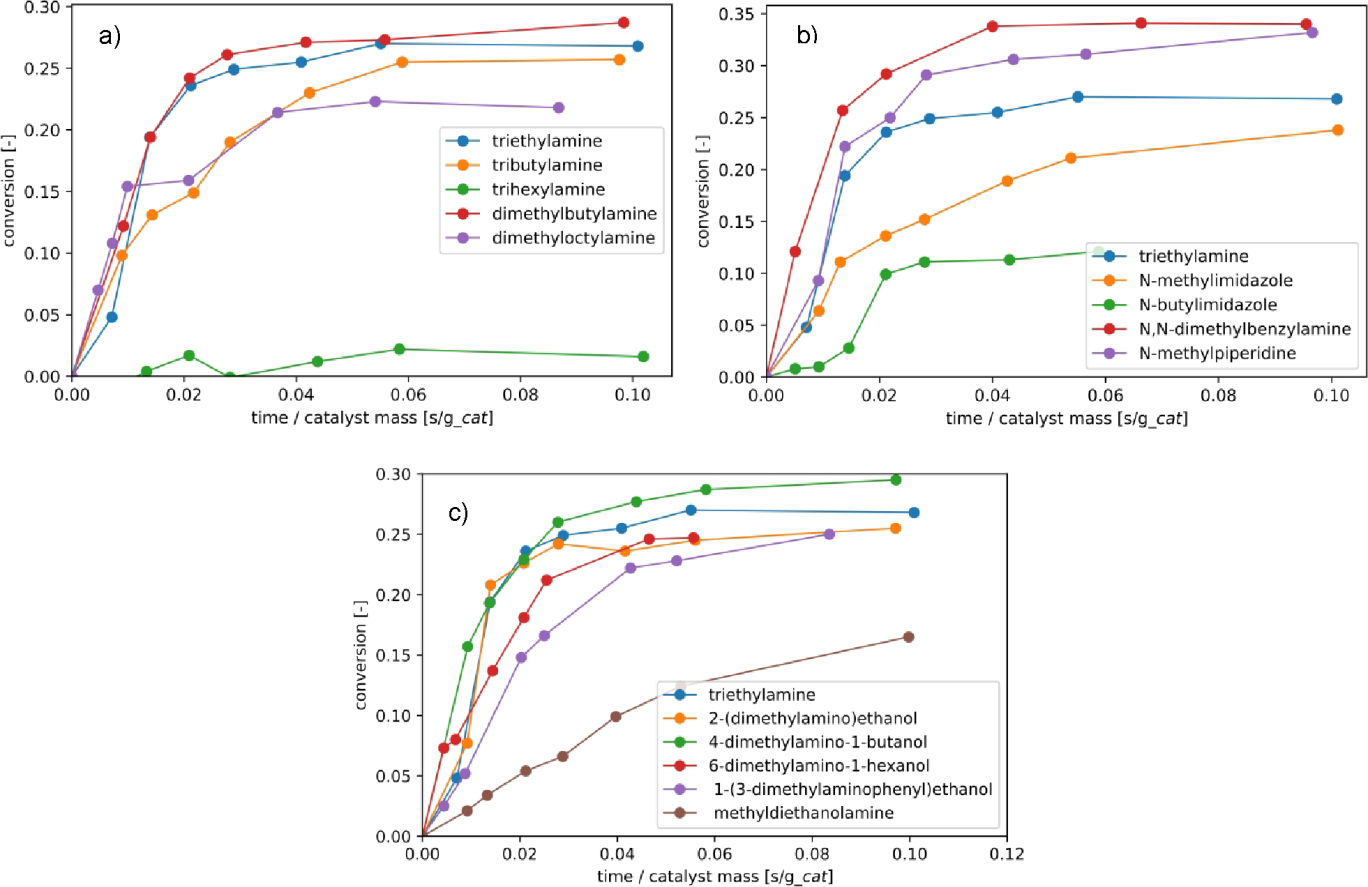
Formic acid conversion versus time for several types of amines in glass batch reactors (20 mL): straight-chain trialkylamines (a), cyclic trialkylamines (b), and alcohol group-substituted trialkylamines (c). Conditions: 1:1 molar ratio FA:amine, with a total formic acid concentration of 1 mol/L using either the alcohol amine or 1-decanol as a diluent. Temperature was 50 °C, catalyst: 10 mg of Au/TiO2. The total batch time was 4 h.

In order to investigate the effect of the tertiary amine used as an extraction base, different formic acid decomposition experiments were carried out in glass batch reactors using Au/TiO_2_ as the catalyst at 50 °C. An overview of the p*K*_a_ and molar volume of all amines is given in the SI. Figure 2a reveals a significant effect of the aliphatic chain length, with kinetic rates decreasing on going from triethylamine to tributylamine to trihexylamine, with the FA conversion being almost negligible in the case of trihexylamine.

While previous work reported a significant dependency between activity and p*K*_a_,^38^ the difference between the p*K*_a_ values of triethylamine (10.225) and trihexylamine (10.318) is negligible. Thus, this decrease in reactivity is likely caused by the bulky alkyl chains, leading to kinetic effects.

To decrease the kinetic diameter of the tertiary amines and, subsequently, the kinetic effects, asymmetrical amines (Figure 2a) and cyclic amines (Figure 2b) were tested. Figure 2a reveals comparable performance in terms of the CO_2_ conversion and kinetic rates between triethylamine (p*K*_a_ 10.225) and dimethylbutylamine (p*K*_a_ 10.191). Extending the alkyl chain to dimethyloctylamine (p*K*_a_ 10.200) results in a decrease in the observed FA conversion. Interestingly, while the reaction time required to reach the equilibrium conversion is comparable between all amines, the equilibrium conversion for the dimethyloctylamine is ∼18% lower. The addition of bases to CO_2_ hydrogenation reactions is known to result in an enthalpy-driven process,^27,41^ with the equilibrium composition being a strong function of the protonation enthalpy gained by formic acid protonating the tertiary amine.

**Figure 3:**
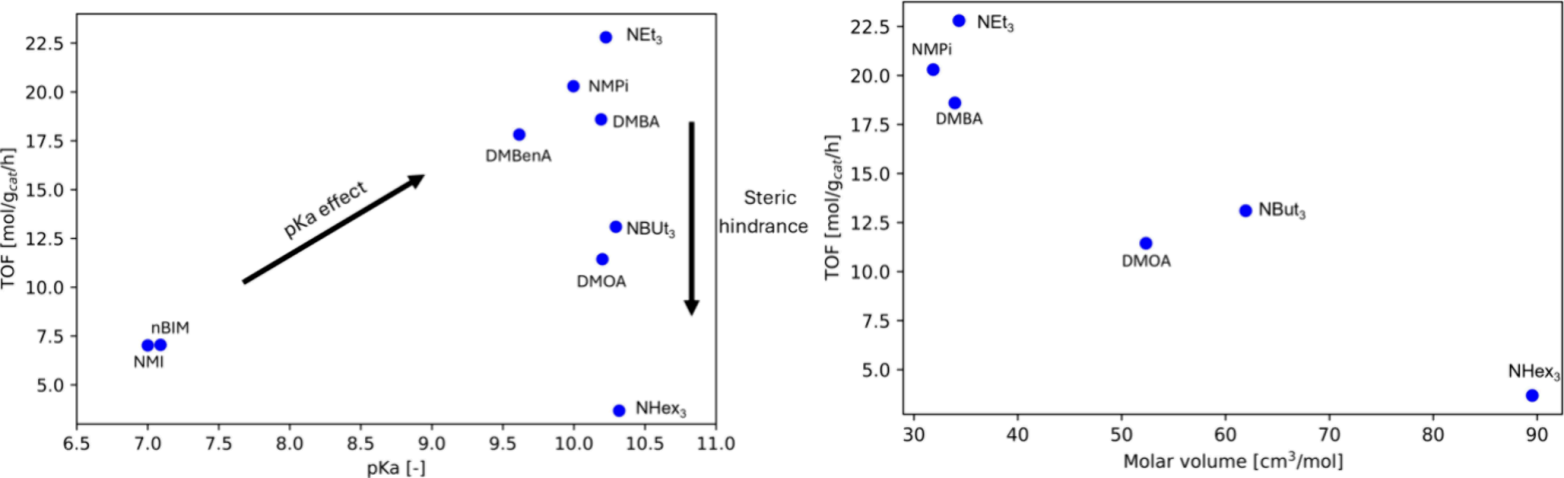
p*K*_a_ versus TOF and molar volume versus TOF observed during FA decomposition over a Au/TiO_2_ catalyst for trialkylamines and cyclic amines. NMI represents *n*-methylimidazole, nBIM *n*-butylimidazole, DMBenA dimethylbenzylamine, NMPi *n*-methylpiperidine, NEt_3_ triethylamine, DMBA dimethylbutylamine, NBut_3_ tributylamine, DMOA dimethyloctylamine, and NHex_3_ trihexylamine.

Three types of cyclic amines were tested, as shown in Figure 2b: piperidines (p*K*_a_ ∼10.1), imidazoles (p*K*_a_ ∼5.1), and cyclic tertiary amines (p*K*_a_ ∼9). Here, FA conversions and kinetics outperforming the benchmark triethylamine were observed for *N*-methylpiperidine and *N*,*N*-dimethylbenzylamine, and imidazoles of several chain lengths result in significant decreases in performance. The p*K*_a_ value of imidazoles is much lower than that of *N*-methylpiperidine and *N*,*N*-dimethylbenzylamine, leading to lower FA conversions. Similar to the case of the alkylamines, an increase in the kinetic diameter caused by an extension of the alkyl chain of the imidazoles leads to a significant decrease in kinetic rates. These effects are visualized within Figure 3, where the TOF versus p*K*_a_ is graphed for all alkyl and cyclic amines. As an activation phase seems to be present for nearly all amines depicted in Figure 2, the TOF values were determined based on the time required to reach equilibrium.

**Figure 4:**
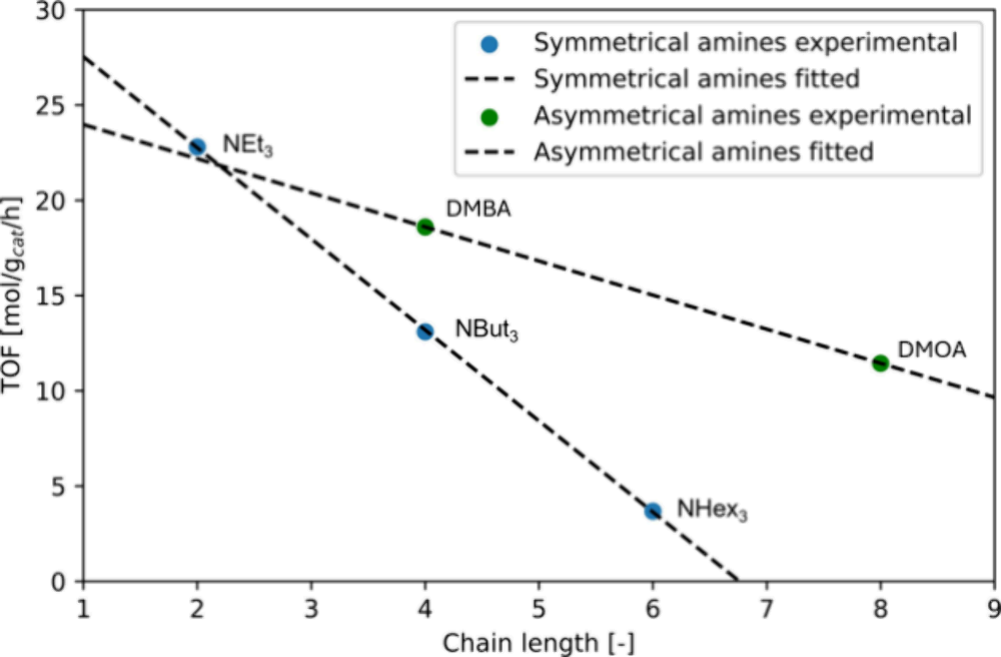
TOF versus alkyl tail length observed during FA decomposition over a Au/TiO_2_ catalyst for trialkyl amines. NEt_3_ represents triethylamine, NBut_3_ tributylamine, NHex_3_ trihexylamine, DMBA dimethylbutylamine, and DMOA dimethyloctylamine.

From Figure 3, two trends can be distinguished, namely, an increase in TOF with an increase in p*K*_a_ for amines of similar molar volumes and a significant decrease in TOF for amines of similar p*K*_a_ but increasing molar volumes. For symmetrical and asymmetrical amines, this trend is observed to be linear, as shown in Figure 4.

From Figure 4, it is found that the slope of the decrease in TOF with an increasing hydrocarbon chain length is much less steep than that in the case for symmetrical amines. This effect is most likely due to the decrease in the volume of the bulky alkyl tails, thus reducing the degree of steric hindrance, of the amine, therefore allowing the formic acid–tertiary amine adducts to diffuse faster through the catalyst pores to reach the catalytic active site.

Filonenko et al. have previously shown the kinetic rate of CO_2_ hydrogenation to formic acid to directly scale with the amine concentration in the reactor,^13^ while Schaub and Paciello^25^ and de Leeuw den Bouter et al.^40^ have demonstrated the necessity of polar solvents. Subsequently, it was hypothesized that the employment of polar tertiary amines could allow for the removal of additional solvents, such as ethanol or 1-decanol, and give rise to enhancement of the kinetic rates. Several OH group-substituted tertiary amines (2-(dimethylamino)ethanol, 6-dimethylamino-1-hexanol, methyldiethanolamine, and triethanolamine) were mixed with formic acid in molar ratios ranging from 0.1 to 10. No phase separation was observed for any of the amines or at any molar ratio, thus allowing for the reaction to proceed without the addition of any additional polar solvents. However, triethanolamine yielded highly viscous, close to solid adducts, which were not suitable for batch or continuous processing.

Based on the results of the alkylamines, it was opted to focus on asymmetrical and cyclic polar amines, with results depicted in Figure 2c. To allow for comparison to the previous experiments, a total formic acid concentration of 1 mol/L was maintained. From Figure 2c, it becomes apparent that the effect of steric hindrance is much less pronounced for asymmetrical amines with one OH group compared to asymmetrical amines without the OH group, with the performance in terms of kinetics and FA conversion of 2-(dimethylamino)ethanol, 4-dimethylamino-1-butanol, and 6-dimethylamino-1-hexanol being very comparable to triethylamine. 1-(3-Dimethylaminophenyl)ethanol yielded lower kinetic rates, most likely due to steric hindrance as the p*K*_a_ value is comparable to the basicity of triethylamine. Lastly, methyldiethanolamine was tested, yielding a very significant decrease in kinetic rates, with no equilibrium achieved after 4 h of the reaction. The methyldiethanolamine–formic acid adducts were observed to be highly viscous, posing a possible explanation for the slower kinetics.

**Figure 5:**
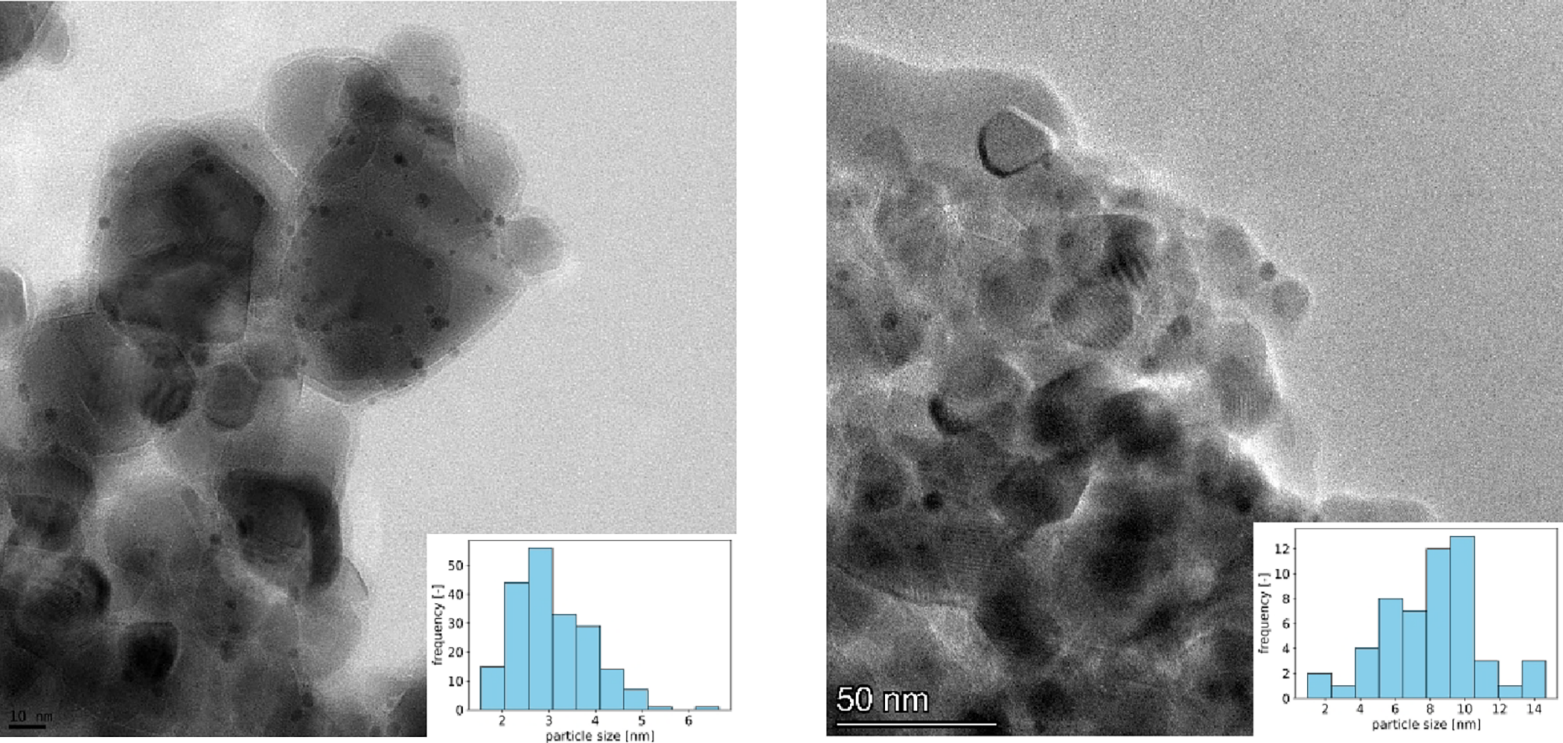
TEM images of a fresh Au/TiO_2_ catalyst (left) and a catalyst exposed to formic acid–triethylamine adducts for 4 h at 50 °C (right). At least 50 particles were analyzed for each sample. The average particle size of the fresh catalyst was confirmed using CO pulse chemisorption, assuming an equal Au/CO chemisorption stoichiometry and hemispherical particles, following the work of Akita et al.^43^

Severe catalyst deactivation was reported for a Pd/C catalyst exposed to formic acid–triethylamine adducts under similar conditions^39^ to the ones used within this study. To ensure stability of the Au/TiO_2_ catalyst, ICP and TEM analyses were performed on the post-mortem samples of the catalyst tested through at least each type of amine. An overview of the obtained results is shown in Table 1. A comparison of the particle size distribution obtained based on TEM measurements for the fresh catalyst and a catalyst exposed to triethylamine– formic acid adducts is shown in Figure 5. All other particle size distributions and exemplary TEM images can be found in the SI (Figure S2).

**Table 1:** Formic Acid Decomposition Post-mortem Particle Size (Obtained from TEM) and Catalyst Loading (Obtained from ICP-OES)^a^

tertiary amine	particle size (TEM) [nm]	catalyst loading [wt %]
triethylamine	4.69 ± 1.84	1.17 ± 0.05
tributylamine	5.78 ± 3.26	1.14 ± 0.03
trihexylamine	5.45 ± 2.22	1.15 ± 0.07
*N*,*N*-dimethylbenzylamine	4.50 ± 1.68	1.17 ± 0.02
2-(dimethylamino)ethanol	4.98 ± 1.74	1.09 ± 0.07
methyldiethanolamine	4.41 ± 1.32	1.11 ± 0.05
6-dimethylamino-1-hexanol	4.32 ± 1.10	1.15 ± 0.03
1-(3-dimethylaminophenyl)ethanol	6.95 ± 1.92	1.10 ± 0.09

a The reaction time was 4 h at 50 degrees Celsius. The initial particle size was established to be 2.6 ± 0.8 nm, and the loading was 1.21 wt %. For the TEM, at least 50 particles were analyzed for each sample. The average particle size of the fresh catalyst was confirmed by using CO pulse chemisorption (2.63 nm).

No significant leaching is observed for any of the amines, while gold nanoparticle sizes increase from 2.6 to 4–7 nm depending on the amine that the catalyst was exposed to. While no significant conversion was observed in the case of formic acid–trihexylamine adducts, a particle size increase from 2.6 to 5.5 nm was observed, leading to the hypothesis that the observed sintering is not caused by the reaction itself but by exposure to formic acid–tertiary amine adducts.

### 3.2 Formic Acid Formation

**Figure 6:**
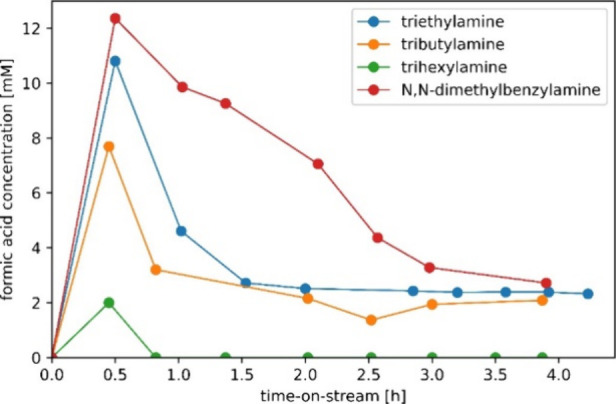
Amine screening of the commercial Au/TiO_2_ catalyst using a continuous flow reactor performing in the CO_2_ hydrogenation mode for several amines. The catalyst was replaced for each test. Employed conditions: 2.5 g of the catalyst, amine:solvent ratio of 1:3 (mol/mol), 70 °C, 40 bar total pressure, and a CO_2_:H_2_ feed ratio of 1:1 at 1 mL/min. The liquid flow rate was 1 mL/min (resulting residence time approximately 10 min).

To validate the usage of the formic acid decomposition reaction as a screening tool to predict the activity of the tertiary amine for formic acid production, triethylamine, tributylamine, and trihexylamine were used in a high-pressure continuous flow packed bed reactor. Experiments were performed under a constant solvent:amine molar ratio and residence time to allow for direct comparison of kinetic rates, and the carbon balance of all experiments was found to be >95%. Figure 6 reveals a trend similar to the one observed in Figure 2 based on the formic acid decomposition reaction with formic acid production decreasing with an increase in the aliphatic chain length on going from triethylamine to trihexylamine. In agreement with the previous observations, the reactive test with trihexylamine resulted in very low to no CO_2_ conversion, while the usage of *N*,*N*-dimethylbenzylamine outperformed triethylamine in terms of productivity. This validates the use of the decomposition reaction for measurement of the kinetic rate and equilibrium composition.

While predictions can be made for the FA productivity, Figure 6 reveals severe catalyst deactivation, which was not expected based on the decomposition study. For the linear chain amines, the rapid deactivation phase of approximately 1.5 h is followed by a constant productivity up to 4 h time-on-stream in a 4 h experiment. The rate of deactivation is much lower for *N*,*N*-dimethylbenzylamine, as it does not reach this stable phase within 4 h time-on-stream.

**Figure 7:**
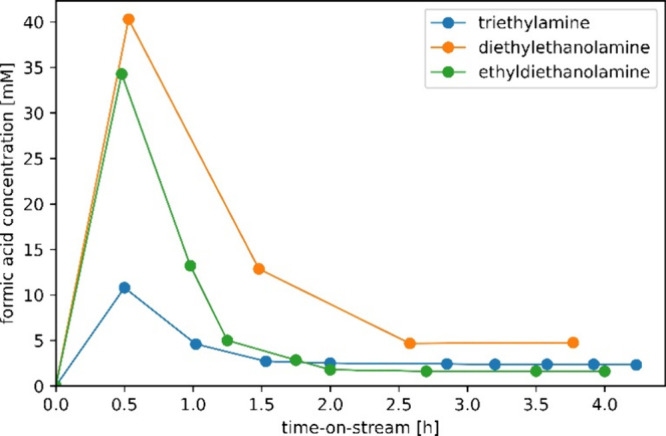
Alcohol amine screening of the commercial Au/TiO_2_ catalyst using a continuous flow reactor performing in the CO_2_ hydrogenation mode, without the usage of additional solvents. The catalyst was replaced each test. Employed conditions: 2.5 g of the catalyst, 70 °C, 40 bar total pressure, and a CO_2_:H_2_ feed ratio of 1:1 at 1 mL/min. The liquid flow rate was 1 mL/min (resulting residence time approximately 10 min).

As shown in Figure 7, a significant increase in initial formic acid production compared to the benchmark triethylamine is observed when polar amines such as diethylethanolamine and ethyldiethanolamine are used, with the FA productivity increasing up to a factor of 5. This increase in productivity is the consequence of no longer requiring the usage of additional solvents such as 1-decanol, resulting in much higher tertiary amine concentrations compared to the case where the amine is diluted within a solvent phase. However, exposure of the Au/TiO_2_ catalyst to diethylethanolamine and ethyldiethanolamine under the studied conditions results in a rapid decrease in productivity, followed by stabilization. Interestingly, the stable formic acid productivity lies in the same concentration range for both triethylamine and ethyldiethanolamine, with the exception of diethylethanolamine. No CO formation was detected during any of the experiments.

To gain a deeper understanding into the deactivation and subsequent stabilization for a longer time-on-stream, two long-duration experiments (1:3 mol/mol triethylamine:1-decanol and diethylethanolamine) were performed in which the catalyst was reused in four separate experiments. Between experiments, the catalyst was kept under argon to prevent air exposure.

**Figure 8:**
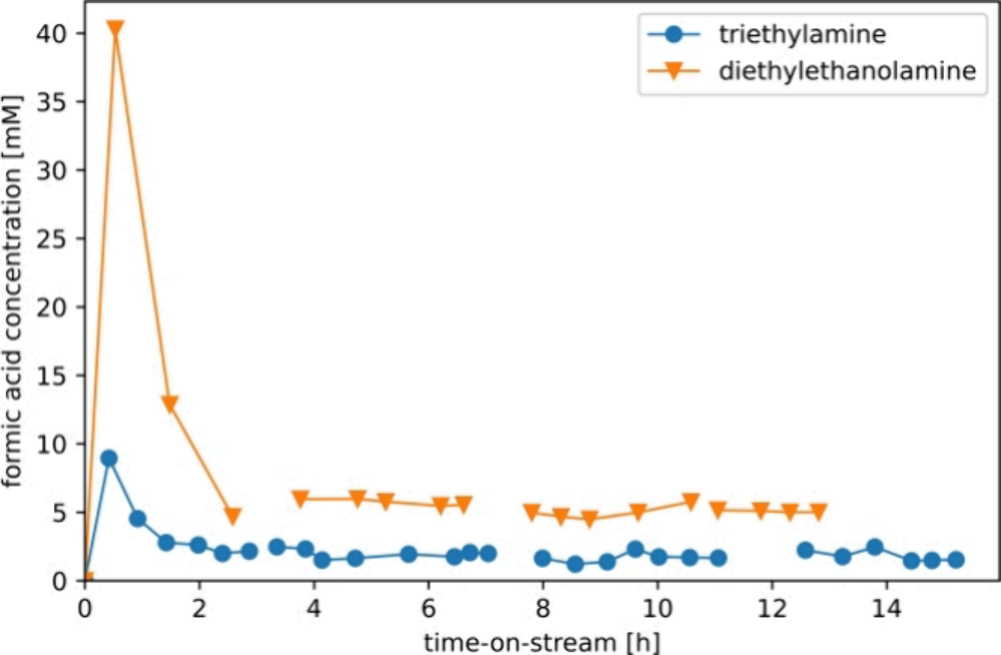
Long-duration stability test of a Au/TiO_2_ catalyst using a continuous flow reactor performing in CO_2_ hydrogenation mode. The catalyst was reused for 4 cycles. Employed conditions: 2.5 g of the catalyst, 70 °C, 40 bar total pressure, and a CO_2_:H_2_ feed ratio of 1:1 at 1 mL/min. The liquid flow rate was 1 mL/min (resulting residence time approximately 10 min).

As shown in Figure 8, the stable FA production rate after the rapid deactivation remains for a longer time-on-stream up to 12 h. The steady-state production of the conventional triethylamine-based process is in the range of 2–3 mM at a residence time of 10 min, while diethylethanolamine results in a nearly double productivity at roughly 5 mM.

Consecutively, the catalysts used for formic acid production (single runs and long-duration tests) were analyzed using a variety of characterization techniques (TEM, ICP-OES, and CO pulse chemisorption). The TEM and ICP-OES results are summarized in Table 2.

**Table 2:** Formic Acid Production Post-mortem Particle Size (Obtained from TEM) and Catalyst Loading (Obtained from ICP-OES)^a^

tertiary amine	particle size (TEM) [nm]	catalyst loading [wt %]
triethylamine (15 min residence time)	12.33 ± 2.91	1.09
tributylamine	7.06 ± 2.52	1.08
trihexylamine	5.50 ± 3.45	1.17
*N*,*N*-dimethylbenzylamine	8.47 ± 3.68	1.13
diethylethanolamine (15 min residence time)	9.14 ± 3.17	1.16
ethyldiethanolamine	5.43 ± 2.32	1.14
triethylamine (4 cycles)	8.76 ± 4.11	1.06
diethylethanolamine (4 cycles)	12.99 ± 3.69	1.19
*n*-butylimidazole (time-on-stream 50 h)	8.57 ± 3.17	1.17

a The reaction time was 4 h at 70 degrees Celsius. The initial particle size was established to be 2.6 ± 0.8 nm and the loading 1.21 wt %. For TEM, at least 50 particles were analyzed for each sample. The average particle size of the fresh catalyst was confirmed using CO pulse chemisorption (2.63 nm).

Similar to the catalysts exposed to formic acid–tertiary amine adducts, no significant leaching is observed for the catalysts used for formic acid production. However, the size of the gold nanoparticles increases from 2.6 up to 13 nm. The catalytic behavior of gold nanoparticles has been shown to be strongly affected by the particle size for several reactions, such as CO oxidation, formic acid decomposition, and the water gas shift reaction.^44^ Loges et al. have demonstrated that the TOFs of the FA decomposition reaction based on an Au/ZrO_2_ catalyst decrease rapidly when increasing the Au particle size from 0.8 nm to negligible at a particle size of 10 nm.^39^

Further catalyst characterization (XPS, XRD, and CO pulse chemisorption) was performed on the catalysts used in the stability test. To eliminate the possibility of TEM-invisible Au species existing on the catalyst surface, low-temperature CO pulse chemisorption was employed at −72 °C. Here, a stoichiometric factor of 1 between CO and Au was used, assuming hemispherical particles.^43^ An example spectrum is included in Figure S7. The average particle sizes obtained were in line with the TEM results, with the determined particle sizes being 2.63, 8.82, and 13.03 nm for the fresh catalyst, four-cycle triethylamine catalyst, and four-cycle diethylethanolamine catalyst, respectively.

XRD measurements were performed to study the crystallographic structure of the fresh and spent catalysts, with the XRD patterns given in SI Figures S5 and S6. No morphological changes were observed.

Filonenko et al. have previously determined the active species during the reaction to be zero-valence Au species.^13^ Thus, the oxidation state of Au on the surface nanoparticles was analyzed using XPS, with the resulting fitting shown in the SI (Figure S4). The spectra of fresh and spent catalysts are presented in the SI (Figure S4). The 4f_7/2_ and 4f_5/2_ Au orbitals were fitted to the obtained data using a peak area ratio of 3:4 between the 7/2 and 5/2 spin orbits.^45^ Peak splitting between these peaks was constrained to be 3.7 eV. A Shirley background and a GL(90) line shape were used, along with a charge correction of adventitious carbon at 248.8 eV.^45−47^ The fits on the spectra indicate that metallic gold was exclusively present on the surface of the catalyst of both fresh and spent catalysts since only Au^0^ without Au^1+^ and Au^3+^ could be fitted to the obtained data.

The significant decrease in FA productivity observed in this work is thus caused by the growth of the Au particles. Yang et al. have reported the sintering of Au nanoparticles supported on titania. During this study, the catalyst was exposed to a 1:1 CO/CO_2_ gaseous mixture, with gold nanoparticles becoming mobile in the range of 300–410 K. It was hypothesized that the movement of Au particles was caused by CO adsorbates.^48^ However, within this work, no CO was detected using in-line GC with a lower detection limit of 2 ppm.

**Figure 9:**
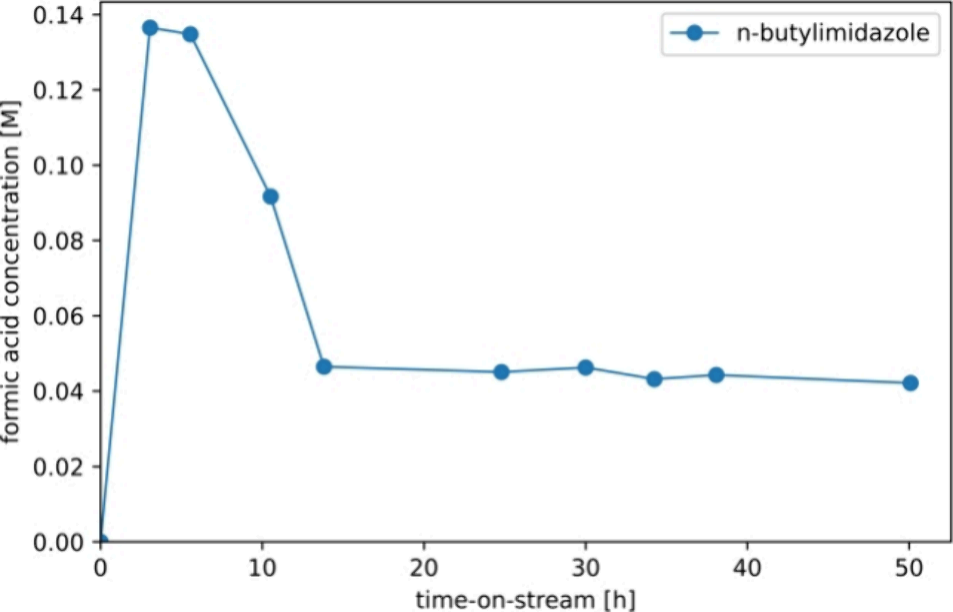
Long-duration testing of *n*-butylimidazole in combination with the commercial Au/TiO_2_ catalyst using a continuous flow reactor performing in CO_2_ hydrogenation mode, without the usage of additional solvents. The catalyst was replaced at each test. Employed conditions: 2.5 g of the catalyst, 70 °C, 40 bar total pressure and a CO_2_:H_2_ feed ratio of 1:1 at 0.73 mL/min. The liquid flow rate was 0.025 mL/min (resulting residence time approximately 2 h).

To investigate if catalyst deactivation also occurs when using less strong bases such as *n*-butylimidazole, long-duration experiments were performed. *N*-Butylimidazole was found to be completely miscible with formic acid in any molar ratio; thus, no additional solvents were required. Experiments with a residence time of 15 min resulted in formic acid productivity below the NMR detection limit. Subsequently, the residence time was increased to 100 min, with the FA production as a function of time-on-stream as shown in Figure 9. Similar behavior is observed as when employing strong bases such as triethylamine, a phase of catalyst deactivation followed by stable FA production of roughly 5 mM. TEM and ICP-OES (Table 2) reveal significant particle growth and no leaching.

## 4 Conclusions

The activity of the direct hydrogenation of CO_2_ toward formic acid was found to strongly depend on the tertiary amine used to reduce the formic acid in the reaction mixture to reduce its activity and thus shift the equilibrium.

Due to the slow kinetic nature of the CO_2_ hydrogenation to formic acid, the formic acid decomposition reaction was used to screen a wide variety of tertiary amines based on the principle of microscopic reversibility. Steric hindrance and the p*K*_a_ of the tertiary amine were identified as key parameters influencing both the observed kinetic rates and the formic acid conversion. Polar tertiary amines allow solventless formic acid production with significantly higher kinetic rates due to higher amine concentrations. Here, the amount of alcohol groups showed a strong influence on the observed kinetic rates, most likely due to viscosity effects.

The assumption of microscopic reversibility was verified in a high-pressure packed bed reactor. Here, the usage of solventless polar amines such as diethylethanolamine allowed for FA productivity up to 5 times that of the benchmark triethylamine system. Catalyst deactivation of the Au/TiO_2_ catalyst was observed for all amines studied within this work, and the deactivation mechanism was shown to be sintering of the Au nanoparticles with no significant leaching, morphological changes, or oxidation of the Au species observed. The catalyst deactivation was found to be very rapid, with the catalyst allowing for stable FA production up to 50 h after the initial gold nanoparticle growth.

## Supporting Information Available

TEM imaging and particle size distribution of all Au/TiO_2_ catalysts, XPS and XRD analysis of spent catalysts, image of the formic acid decomposition setup, confirmation of the absence of mass transfer limitations during experiments, and the molecular structure and p*K*_a_ of all studied amines (PDF)
