# Supplementary material for: Effect of Tertiary Amine Selection on CO2 to Formic Acid Hydrogenation with the Au-np Catalyst
Source: Ind Eng Chem Res. 2025 Apr 10;64(16):8109–18. doi: 10.1021/acs.iecr.4c04902 (PMC12022983; doi:10.1021/acs.iecr.4c04902)
Supplement: Supplementary file 1 — ie4c04902_si_001.pdf [file ie4c04902_si_001.pdf]

Supporting information

Effect of tertiary amine selection on CO_2 to formic acid hydrogenation with Au-np catalyst

Anouk W.N. de Leeuw den Bouter[1,2], Luca M.P. Meijer[1], Larissa Brito[3], Adeline Miquelot[2], Pierre Olivier[2], John van der Schaaf[1*]

1) Sustainable Process Engineering, Chemical Engineering and Chemistry, Eindhoven University of Technology, Het Kranenveld 14, 5612 AZ Eindhoven, The Netherlands

2) Lab Hydrogen, ENGIE Lab CRIGEN, 4 RUE JOSEPHINE BAKER , Stains,N/A France 93240

3) Lab Biogas, Biomass and Waste, ENGIE Lab CRIGEN, 4 RUE JOSEPHINE BAKER , Stains,N/A France 93240

*Corresponding Author, j.vanderschaaf@tue.nl

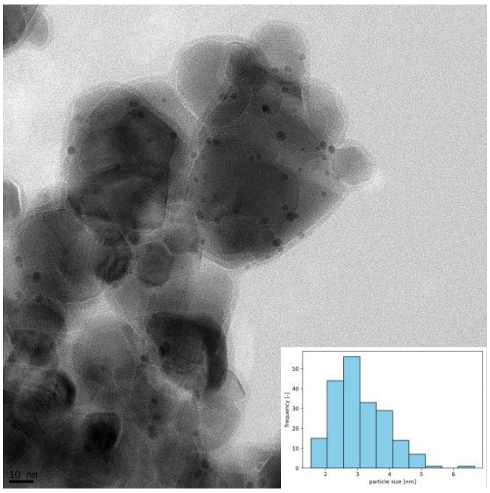

fresh Au/TiO_2

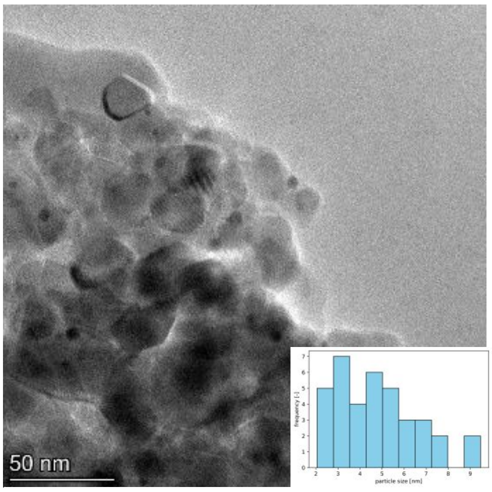

Triethylamine

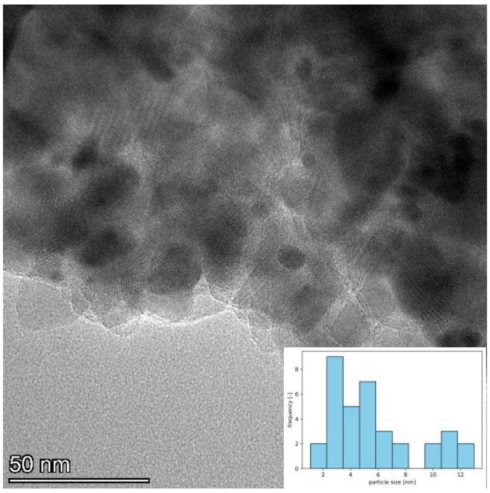

Tributylamine

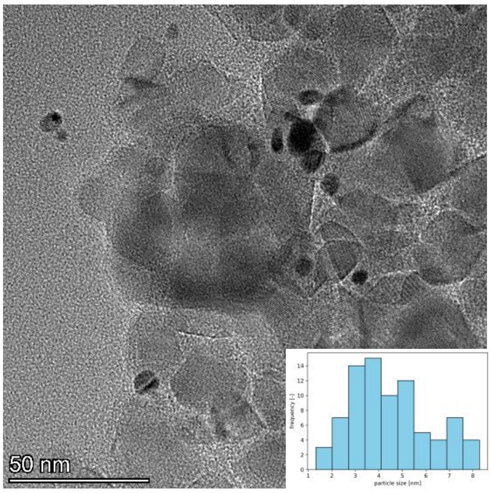

Trihexylamine

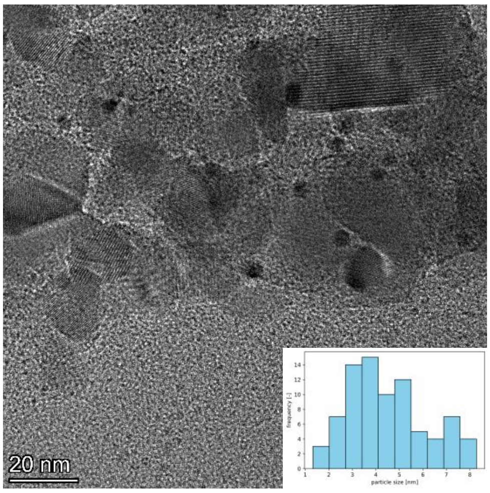

N,N-dimethylbenzylamine

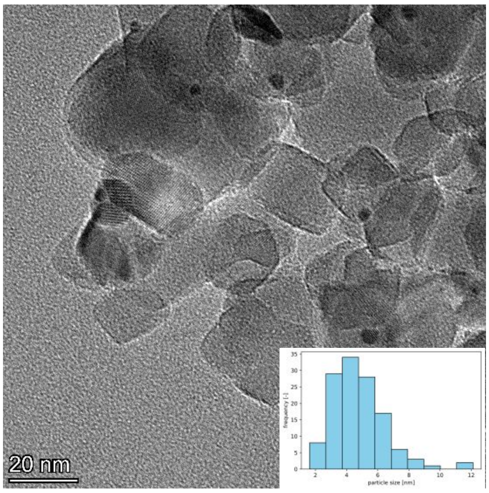

2-(dimethylamino)ethanol

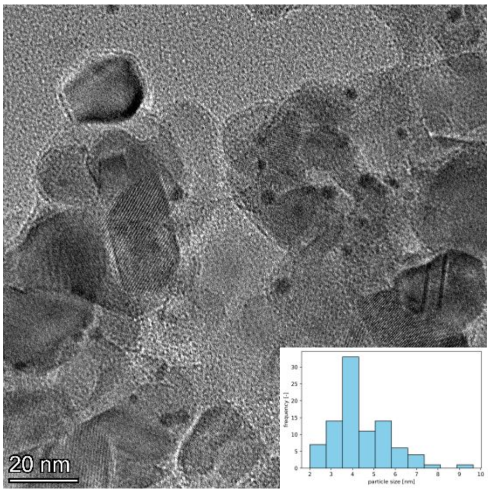

Methyldiethanolamine

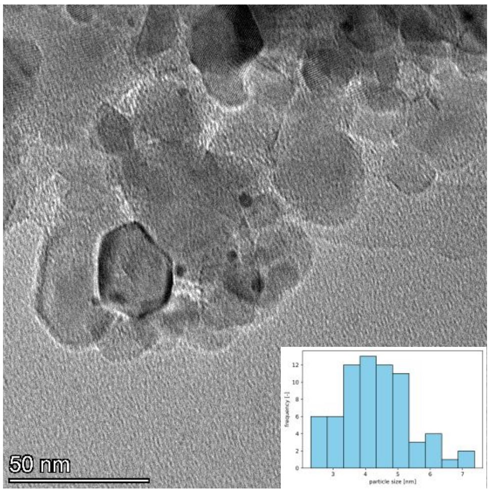

6-dimethylamino-1-hexanol

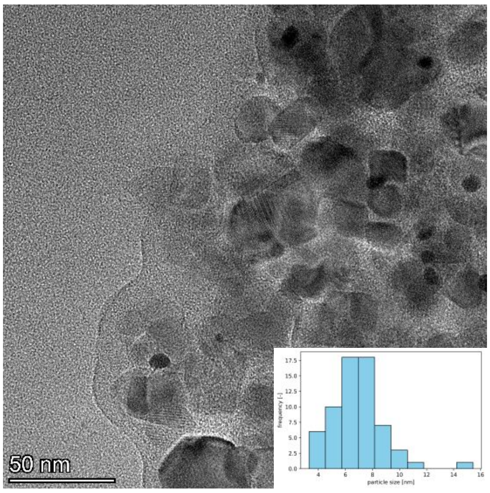

1-(3-dimethylaminophenyl)ethanol

Figure S1: TEM images and particle size distributions of Au/TiO_2 catalysts used in formic acid decomposition. Reaction time was 4 hours at 50 degrees. At least 35 particles were analyzed per sample

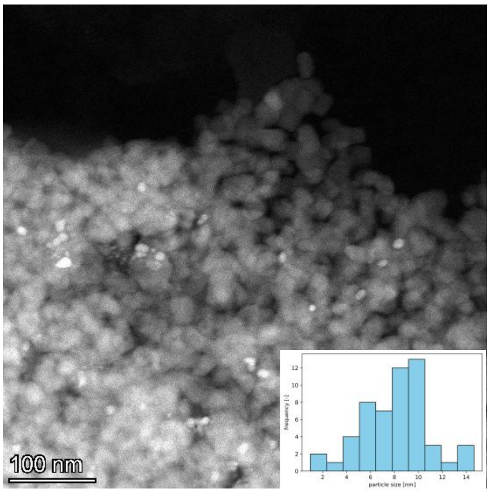

Triethylamine

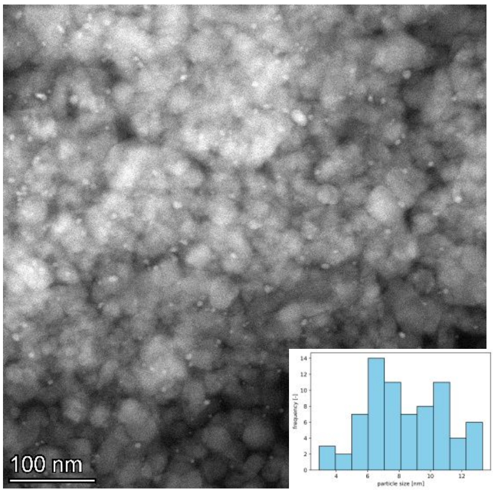

Tributylamine

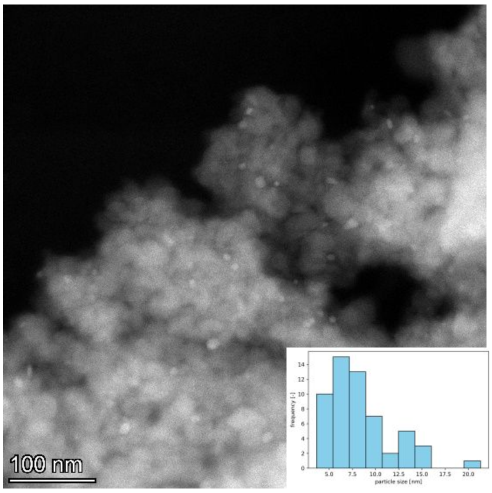

Trihexylamine

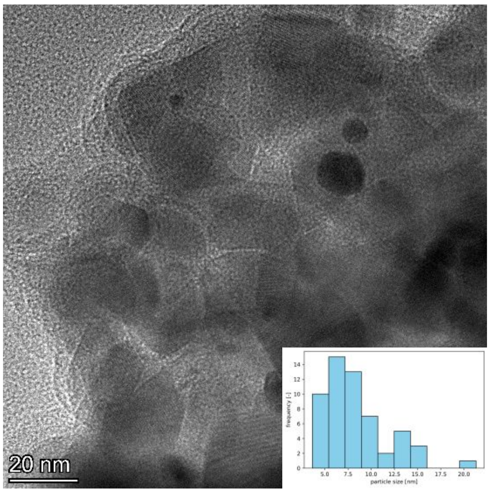

Diethylethanolamine

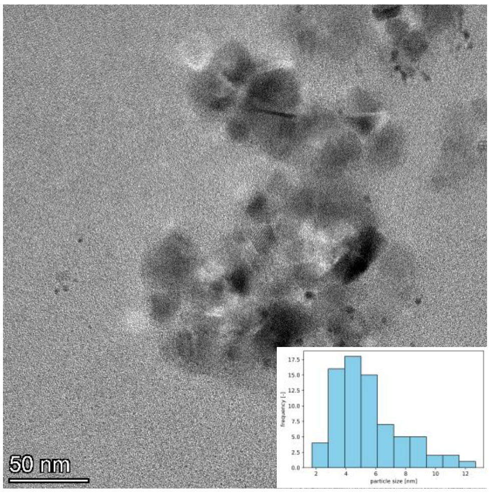

Ethyldiethanolamine

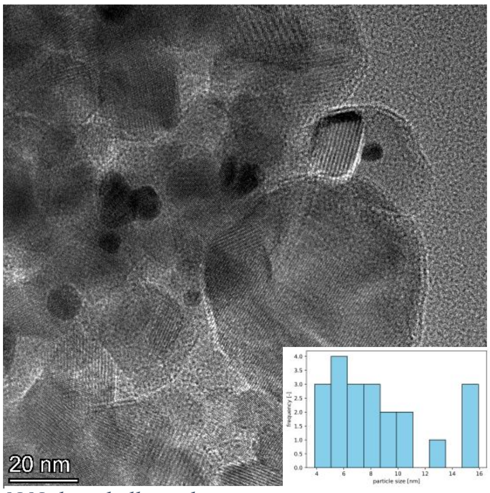

N,N-dimethylbenzylamine

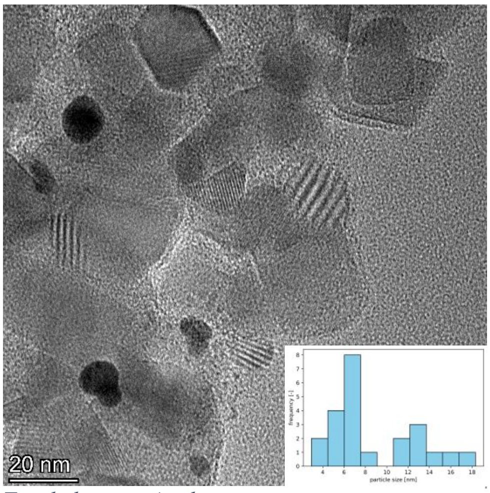

Triethylamine, 4 cylces

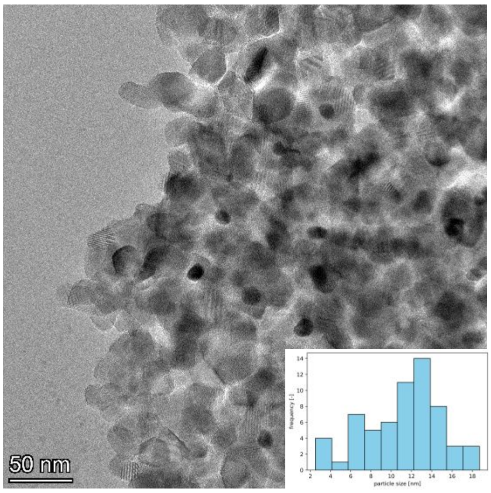

Diethylethanolamine, 4 cylces

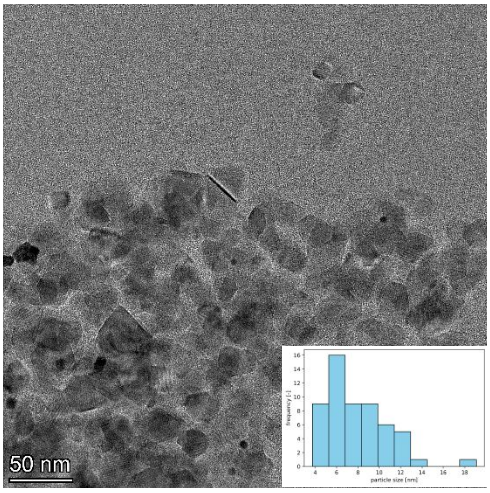

N,N-dimethylbenzylamine, 72hr reaction

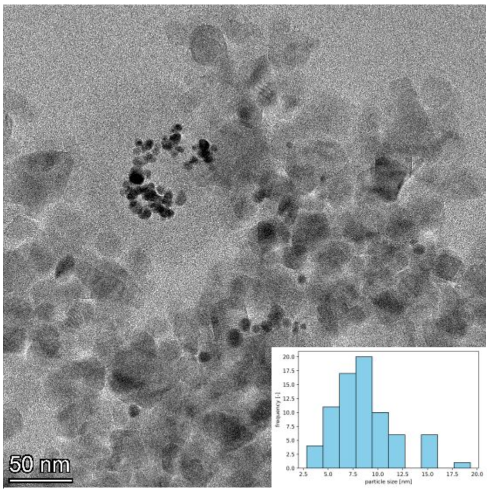

N-butylimidazole, 72hr reaction

Figure S2: TEM images and particle size distributions of Au/TiO_2 catalysts using in the direct hydrogenation of CO_2 to formic acid . Reaction time was 4 hours at 70 degrees, unless otherwise indicated. At least 50 particles were analyzed per sample

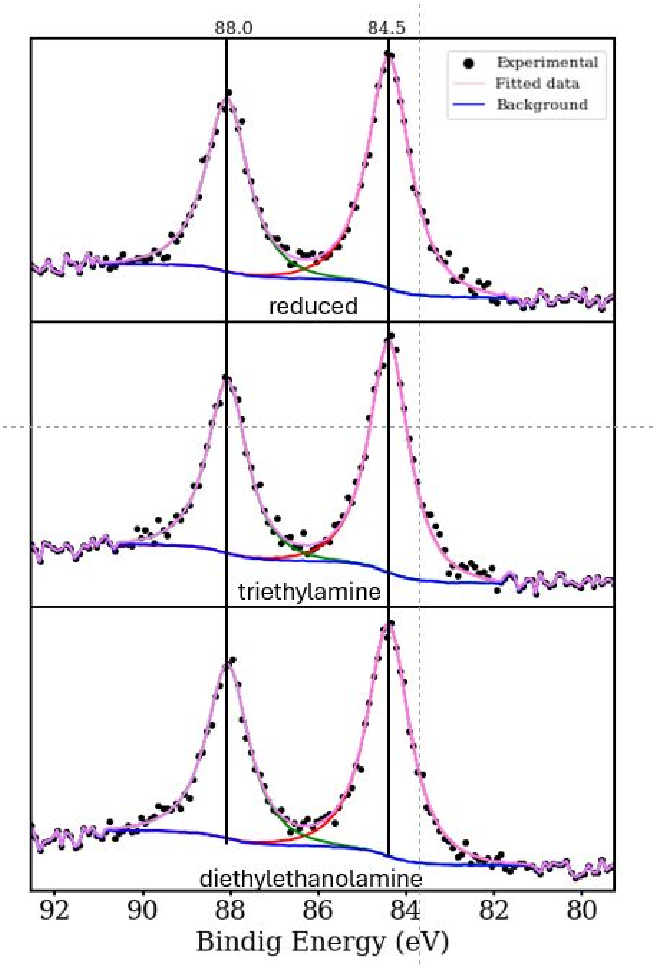

Figure S3: XPS spectra of the Au 4f orbital of fresh Au/TiO_2, spent Au/TiO_2 exposed to 1:3 mol/mol triethylamine: 1-decanol for 12 hours time-on-stream and spent Au/TiO_2 exposed to diethylethanolamine for 12 hours time-on-stream

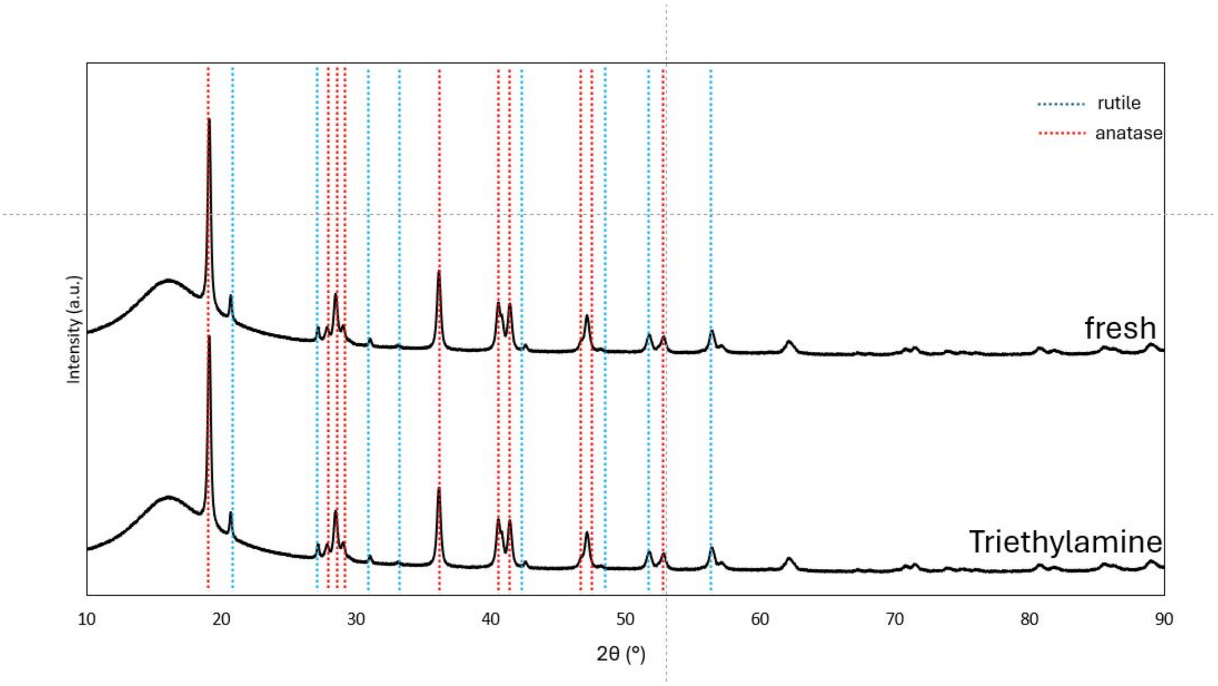

Figure S4: XRD pattern of fresh Au/TiO$_2$ and spent Au/TiO$_2$ exposed to 1:3 mol/mol triethylamine: 1-decanol for 12 hours time-on-stream

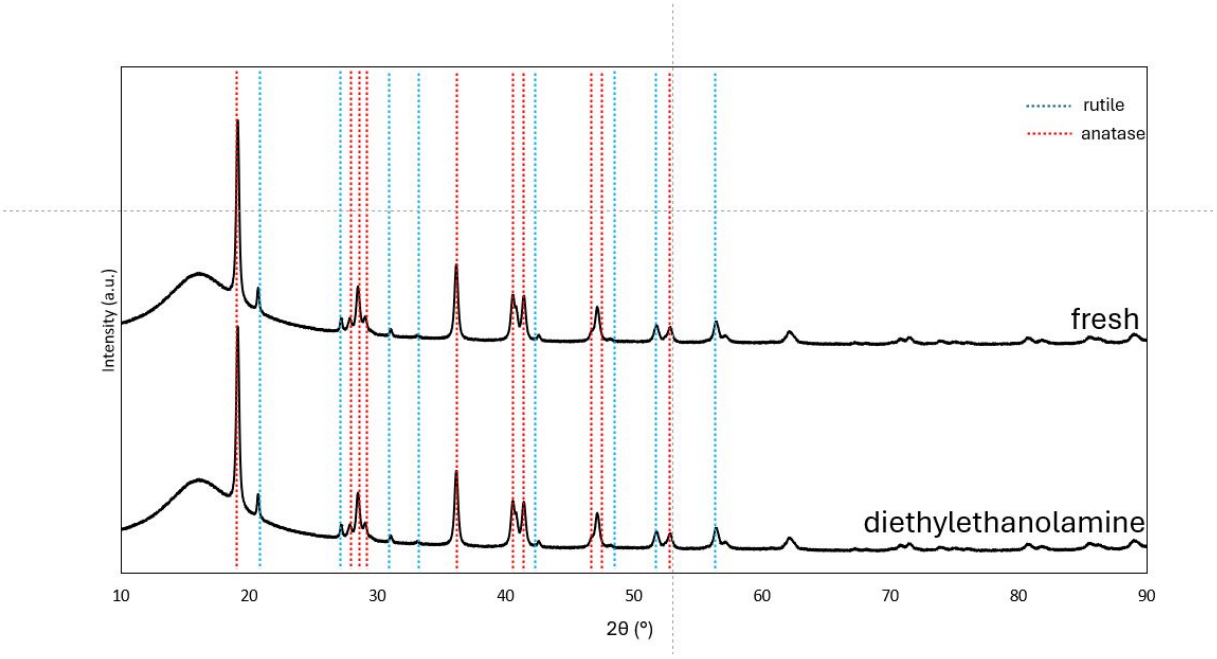

Figure S5: XRD pattern of fresh Au/TiO$_2$ and spent Au/TiO$_2$ exposed to diethylethanolamine for 12 hours time-on-stream

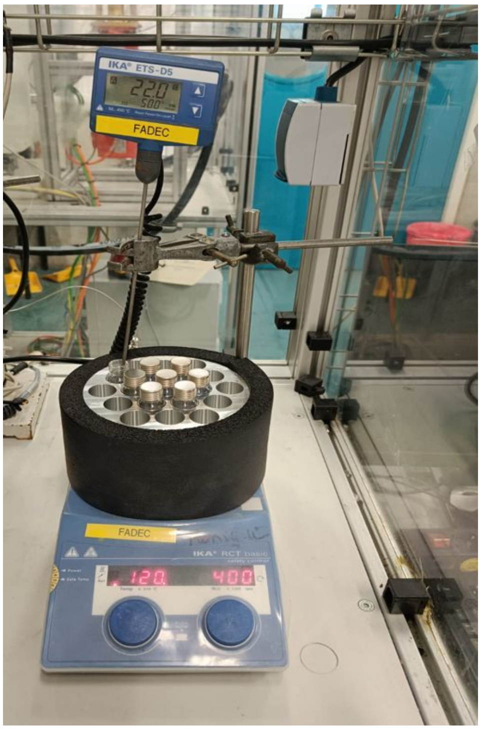

Figure S6: Set-up for batch formic acid decomposition

External Diffusion: *Mears Criterion*

The absence of external mass transfer limitations can be evaluated using the Mears criterion [1]:

$$w = \frac{r_{obs} \cdot \rho_B \cdot R_p}{k_{ls} \cdot C_{Ab}} \leq 0.15$$

Here, r_{obs} represents the observed reaction rate per kilogram of catalyst, ρ_B the bulk density of the catalyst bed, R_p the catalyst particle size, k_{ls} the external mass transfer coefficient and C_{Ab} the bulk concentration. The bulk density of the catalyst was determined by

$$\rho_B = \frac{\rho_{TiO_2}}{1 - \varepsilon_{void}}$$

The mass transfer coefficient was determined using a mass transfer coefficient correlation for small laboratory batch reactors by Pestre *et al*. [2]:

$$k_{ls}a_{ls} = 9.5 \cdot 10^{-4} \, N^{2.2}$$

Here, N is the stirring speed. The Mears criteria were calculated for all reactions, however, the only the formic acid decomposition reaction with dimethylbenzylamine (highest observed reaction rate) is illustrated here.

$$w = \frac{r_{obs} \cdot \rho_B \cdot R_p}{k_{ls} \cdot C_{Ab}} = 0.011 \leq 0.15$$

It can thus be concluded that the system does not suffer form external mass transfer limitations for any of the reactions.

Internal diffusion: *Weisz-Prater Criterion*

The absence of internal mass transfer limitations was evaluated using the Weisz-Prater criterion [1]. Here, internal mass transfer limitations are absent when:

$$C_{wp} = \frac{-r_{obs} \cdot R_p^2}{D_e \cdot C_{As}} \leq 3\beta$$

Here, r_{obs} is the observed volumetric reaction rate, R_p the catalyst particle size, D_e the effective diffusivity and C_s the reactant concentration at the particle surface. β is described by:

$$\beta = \frac{4\,(1 - \eta)}{n}$$

where η and n are the effectiveness factor and reaction order respectively. The effectiveness factor was determined by the Thiele equation for spherical particles:

$$\eta = \frac{\tanh\varphi}{\varphi}$$

$$\varphi = \sqrt{\frac{2k^{-} \cdot R_p^2 \cdot C_0^{n-1}}{D_e}}$$

and,

$$D_e = \frac{\varepsilon_{void}}{\tau} \cdot D$$

With τ the tortuosity, which was taken equal to 3. The diffusivity was taken equal to 1e-6.

$$\varphi = \sqrt{\frac{2k^{-} \cdot R_p^2 \cdot C_0^{n-1}}{D_e}} = \sqrt{\frac{2 \cdot 6.88 \cdot 10^3 \cdot \exp\left(\frac{-31200}{8.314 \cdot 373}\right) \cdot (37.5 \cdot 10^{-6})^2 \cdot 1000^2}{\frac{0.492}{2.93} \cdot 10^{-6}}} = 2.22$$

$$\frac{-r_{obs} \cdot R_p^2}{D_e \cdot C_{As}} \leq 3\,\frac{4\,(1 - \frac{\tanh\varphi}{\varphi})}{n} \rightarrow 0.0066 \leq 6.72$$

It can thus be concluded that the system does not suffer form internal mass transfer limitations for any of the reactions.

Molecular structures and pKa

Structure	pKa	Moleculair volume [cm^3/mol]
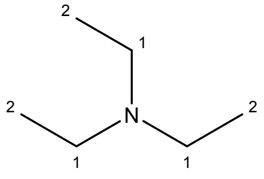	10.225	34.34
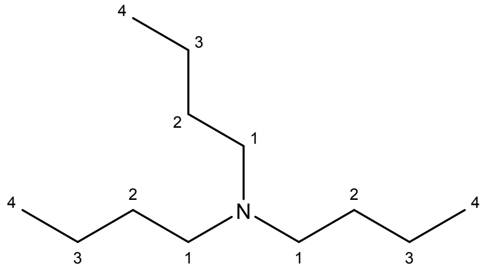	10.295	61.94
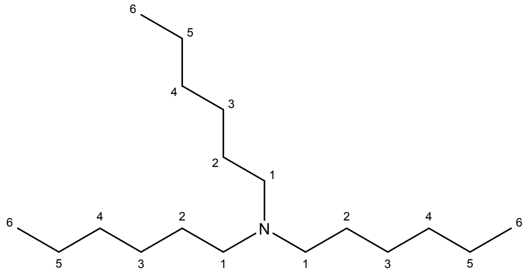	10.318	89.53

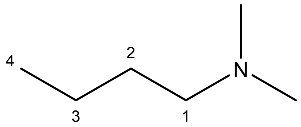 dimethylbutylamine	10.191	33.94
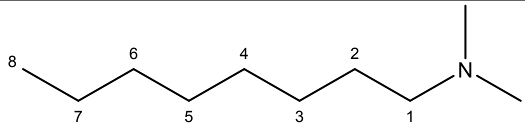 dimethyloctylamine	10.200	52.34
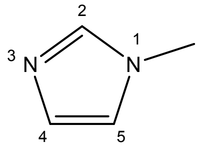 N-Methylimidazole	7.0	24.34
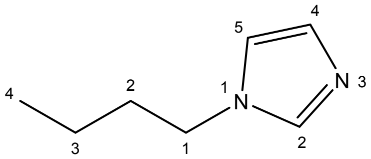 N-butylimidazole	7.09	38.34
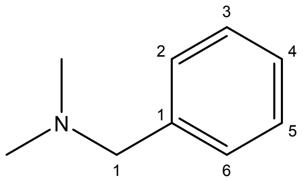 dimethylbenzylamine	9.615	44.44
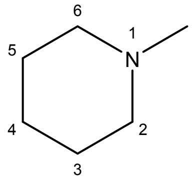 N-Methylpiperidine	9.996	31.86
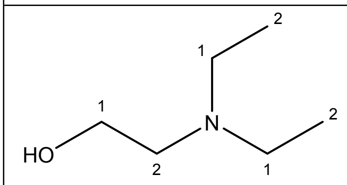 diethylethanolamine	9.389	35.71

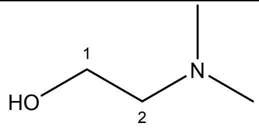<br>Dimethylethanolamine	9.331	26.11
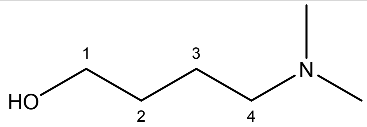<br>Dimethylbutanolamine	10.002	35.31
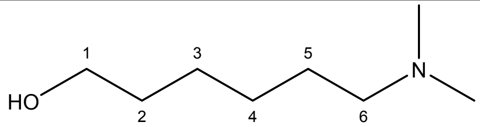<br>dimethylhexanolamine	10.176	44.50
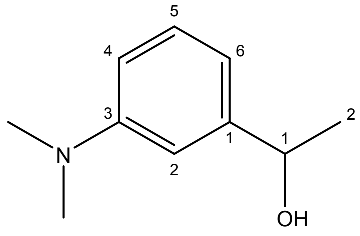<br>1-(3-Dimethylaminophenyl)ethanol	14.373	52.41
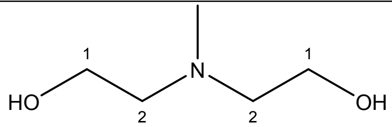<br>Methyl diethanolamine	8.524	32.27
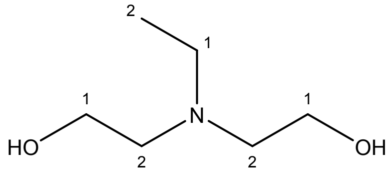<br>ethyldiethanolamine	8.553	35.71

Table S1: pKa and molecular volume for all studied amines. Values were predicted using Chemdraw Professional 18.0

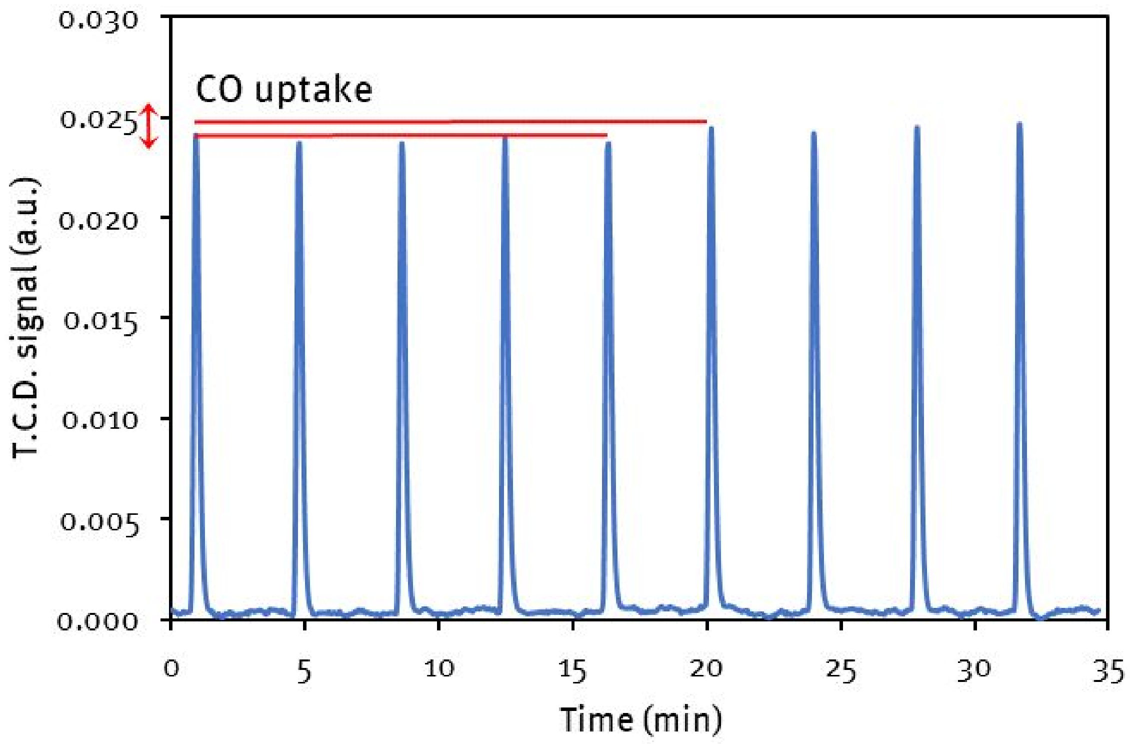

Figure S7: Example plot of the TCD signal versus time obtained during CO pulse chemisorption at -75 °C.

References

[1] Fogler, H. Scott. Elements of Chemical Reaction Engineering. Upper Saddle River, N.J. :Prentice Hall PTR, 199.

[2] V.Meille, N. Pestre, P. Fongarland, and C. De Bellefon, "Gas / Liquid Mass Transfer in Small Laboratory Batch Reactors : Comparison of Methods," *Ind. Eng. Chem. Res.*, vol. 43, pp. 924–927, 2004.
